# A Case Report and Literature Review on the Management of Foreign Body-Related Duodenal Perforation: Can We Avoid the Scalpel?

**DOI:** 10.7759/cureus.81534

**Published:** 2025-03-31

**Authors:** Samuel Ho Ting Poon, Lok Hin K Law, Tat Ming D Chung

**Affiliations:** 1 Department of Surgery, Pamela Youde Nethersole Eastern Hospital, Hong Kong Island, HKG

**Keywords:** duodenal perforation, emergency surgery, non operative management, swallowed foreign body, upper gastrointestinal endoscopy (ogd)

## Abstract

Gastrointestinal perforation remains one of the most commonly encountered surgical emergencies. Despite the advancement in surgical techniques and treatment modalities, retroperitoneal duodenal perforation remains a lethal surgical emergency. While perforations of the gastrointestinal tract are typically managed with laparotomy for repair and decontamination, the advancement in endoscopic technique and equipment shed a light on non-operative management for intestinal perforation. We present here a case of duodenal perforation successfully treated by endoscopic approach.

## Introduction

Gastrointestinal perforation remains one of the most commonly encountered surgical emergencies. Among the causes of perforation, foreign body-related perforation is rare but associated with significant mortality [1]. Despite the advancement in surgical techniques and treatment modalities, retroperitoneal duodenal perforation remains a lethal surgical emergency with mortality up 30% [2]. While perforations of the gastrointestinal tract are typically managed with laparotomy for repair and decontamination, operations for foreign body-related retroperitoneal duodenal perforation are technically challenging and associated with inescapable morbidities due to difficult accesses and infection of the operative field. The advancement in endoscopic technique and equipment shed a light on non-operative management for intestinal perforation [3,4]. We present here a case of duodenal perforation caused by foreign body ingestion, which was successfully treated by endoscopic approach without any morbidity.

## Case presentation

Presentation

**Figure 1:**
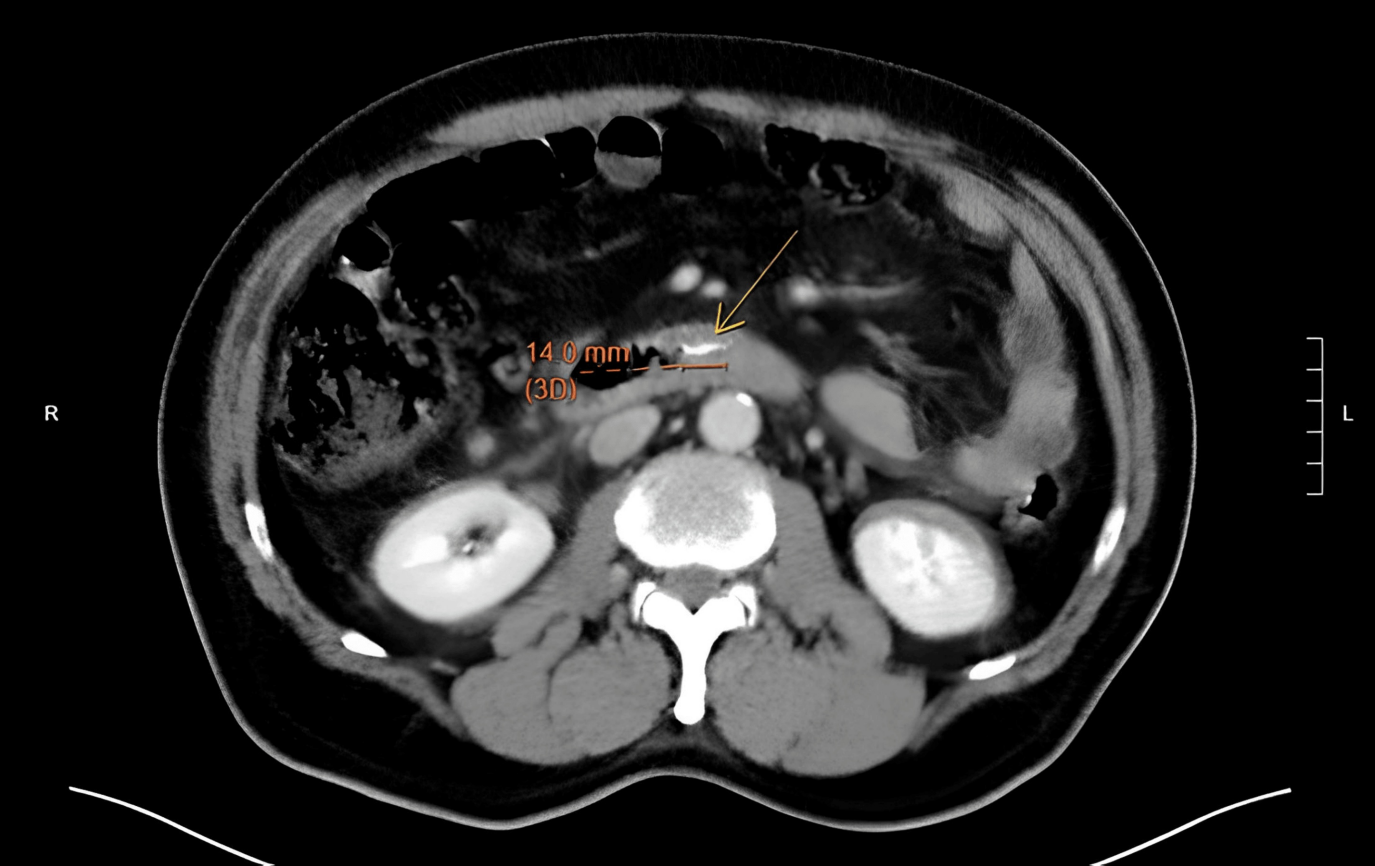
CT film showing a 1.4cm foreign body in the D3 region (annotated)

**Figure 2:**
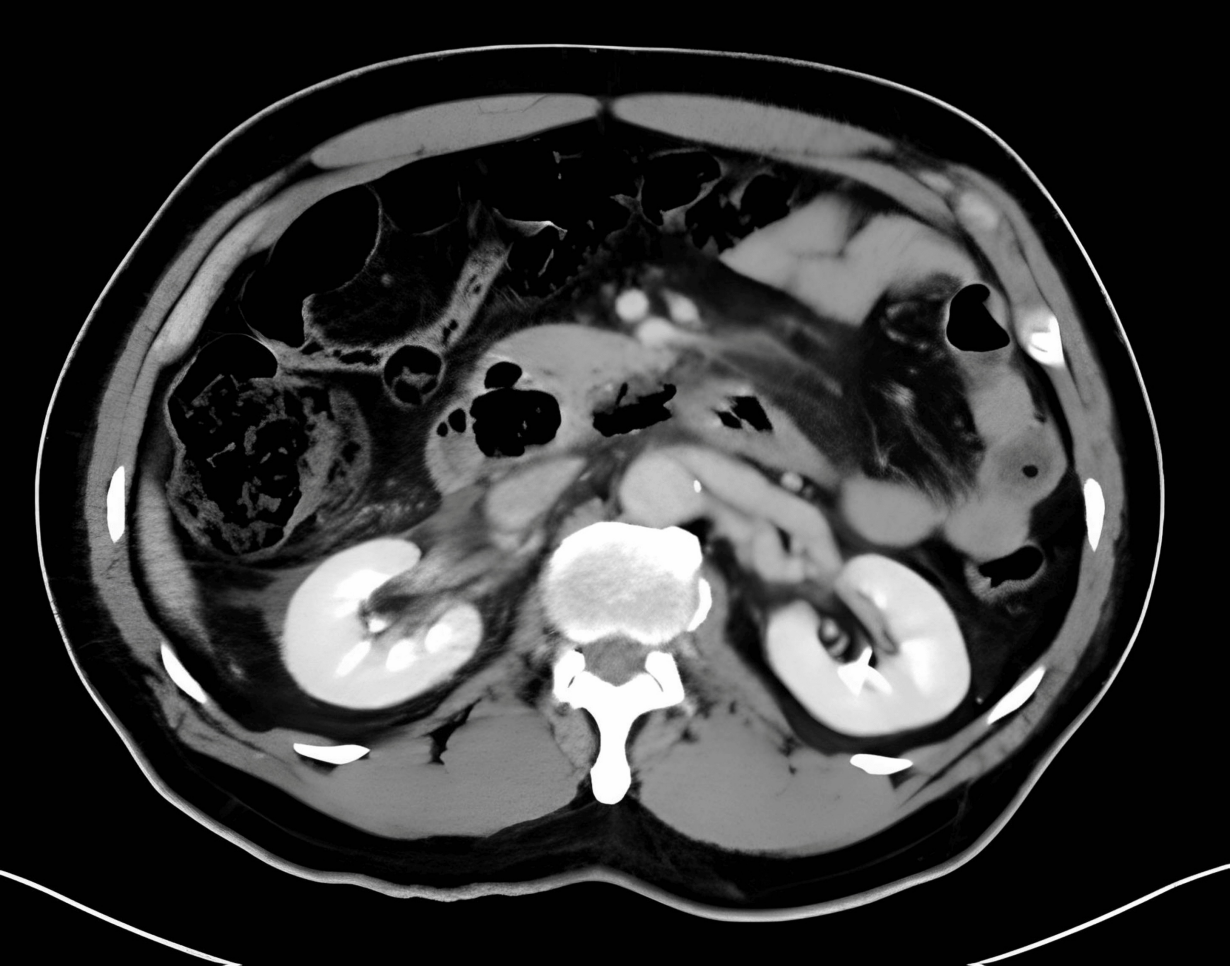
CT film demonstrating perforation of duodenum with retroperitoneal free gas

A 65-year-old gentleman presented to the Emergency Department in the evening for a sudden onset epigastric pain, associated with vomiting of undigested food. There was no symptom of gastrointestinal bleeding. No history of foreign body ingestion was reported. He had a fever of 38.4°C which promptly subsided. Otherwise, all vital signs including blood pressure and pulse were normal. Physical examination found significant epigastric tenderness with rigidity. Chest and abdominal radiography showed no feature of perforated hollow viscus. Blood tests showed an elevated white blood cell count of 16.6x10^9^/L, while other parameters remained unremarkable. A contrast computed tomography of the abdomen was arranged to rule out concealed perforation of the GI tract given the clinical findings. The scan revealed a 1.4cm curvilinear foreign body impacted at the third part of duodenum, with surrounding retroperitoneal free gas, suspicious of duodenal perforation (Figure 1 & Figure 2).

Management

The patient was haemodynamically stable and not in overt sepsis. The perforation was confined to the retroperitoneal cavity on CT with no free fluid within the peritoneal cavity. The location of the foreign body appeared in between the superior mesenteric artery (SMA) and aorta. Surgical management seemed to be technically difficult requiring extensive mobilization of the intestinal tract. We therefore offered an attempt of endoscopic management to the patient.

**Figure 3:**
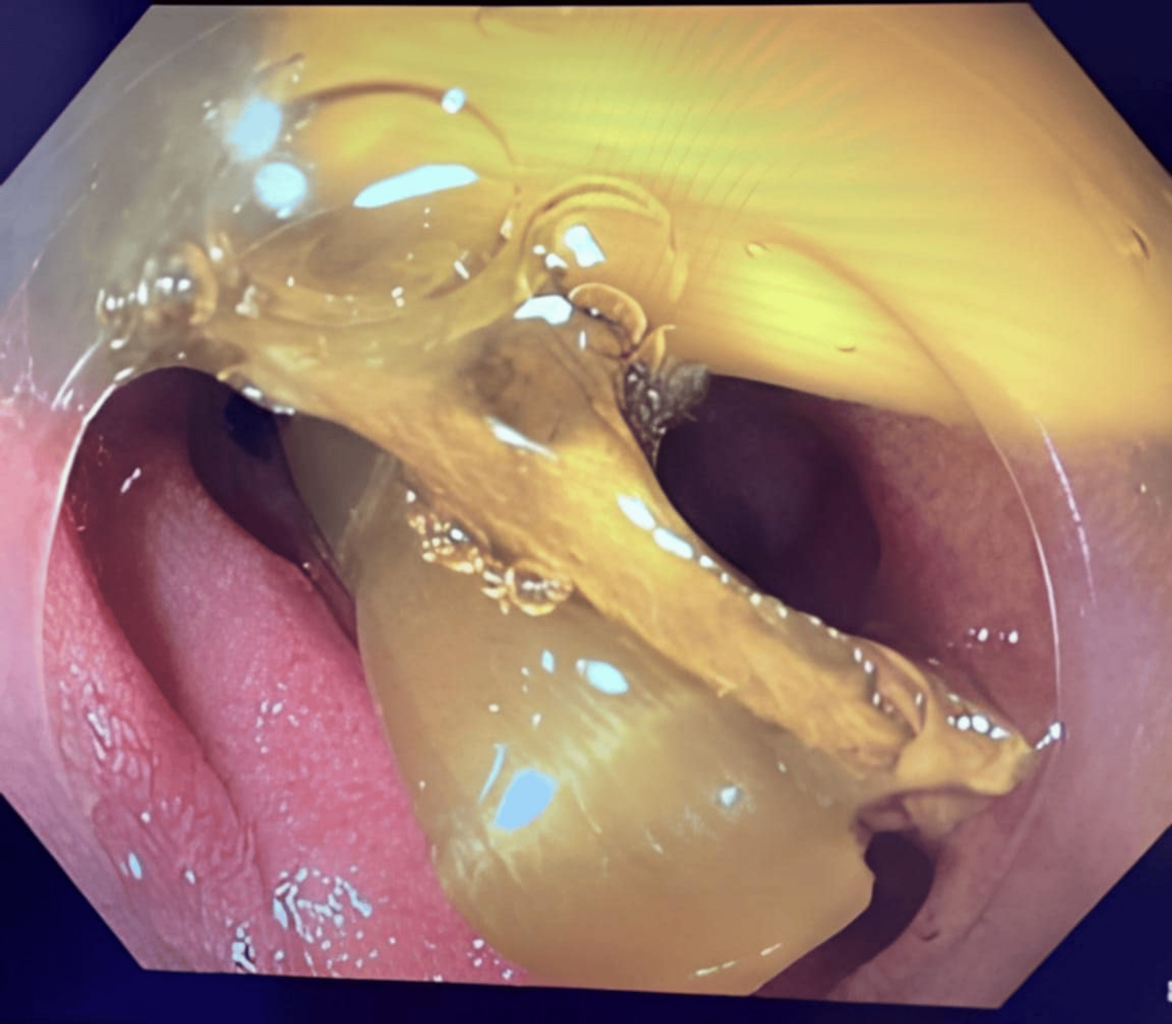
A piece of fish bone impacted on the wall of D3

**Figure 4:**
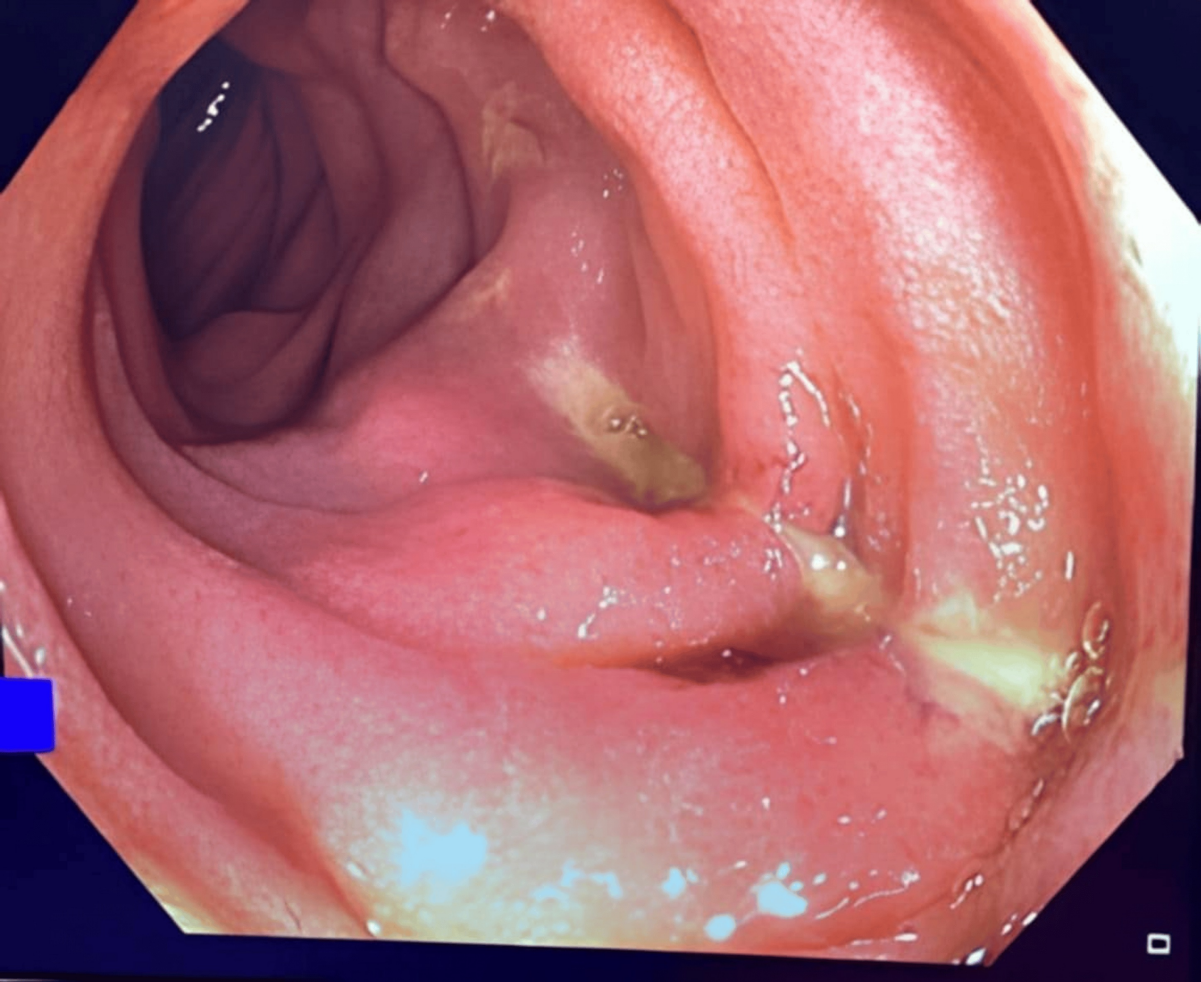
A linear ulcer with a small perforation site was found after removal of the fish bone

**Figure 5:**
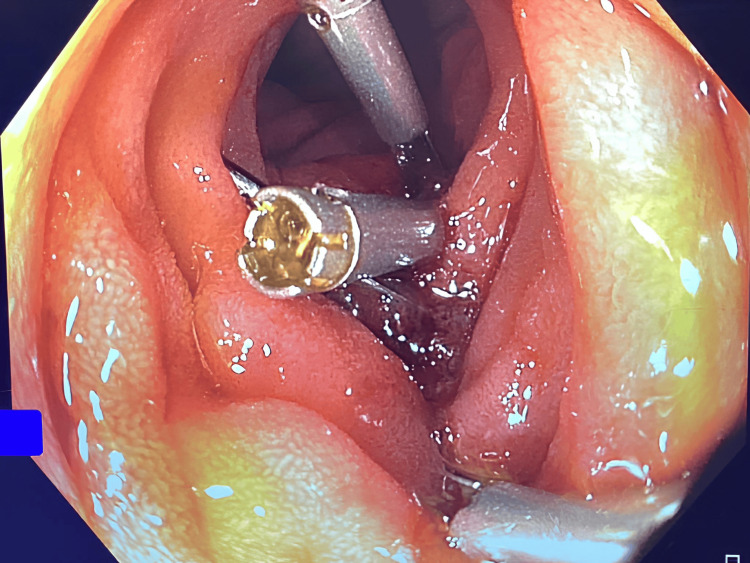
Endoscopic view of closure of the ulcer with endoclips

Under general anaesthesia, the procedure started with 2T-gastroscope. A 2.7cm pentagonal fish scale was found at D3 causing a linear ulcer over the infero-posterior side of the duodenum. The scale impacted firmly and deemed unsafe for direct removal. Endoscopic rat tooth forceps were applied and the scale was cracked into three pieces. Overtube was applied with the fragments retrieved. Reassessment of the ulcer found a 1mm perforation at the deepest part of the ulcer accountable for the retroperitoneal gas (Figures 3-5). The ulcer and the hole were obliterated with three metal clips. Further assessment of proximal jejunum ~60cm from DJ flexure was performed with standard colonoscope and confirmed there was no mucosal injury nor foreign body. The procedure was concluded with nasogastric tube insertion to the stomach for decompression via endoscopic guidance.

The patient was put on antibiotics and antifungal agent. He remained stable after retrieval of the foreign body with fever subsided on post-procedure day 3. An oral contrast CT scan was performed, showing no contrast leakage around the duodenum one week following the procedure. The patient was then resumed on the oral diet and discharged home 13 days after endoscopy.

## Discussion

Management of duodenal perforation remains challenging given its difficult anatomical position and high mortality. Primary closures of the perforation are not always feasible in view of contamination and delayed perforation in which diversion and drainage are required to allow proper healing. To formulate a definite management plan, an extensive kocherization lays the foundation of success by providing a proper exposure for assessment and adequate drainage of associated collections. Nonetheless, this mobilization will breach the concealed retroperitoneal plane to form a communication with the peritoneal cavity and this may result in severe sepsis in case of leakage from the repair site. In addition, retrieval of a large intraluminal foreign body requires further extension of the perforation and this extends the insults and further compromises the healing of the duodenum.

In the past decades, the advancements in endoscopic devices shed a new light to the management of concealed luminal perforation. Several individual case reports have illustrated the feasibility of endoscopic repair of duodenal perforation caused by foreign bodies. Various devices, including the over-the-scope-clips and the endoscopic negative pressure therapy, allow endoscopic closure of large duodenal perforation and drainage of the extraluminal collection associated with the insult [5-8].

However, no organised systematic review or meta-analysis has been done to evaluate its efficacy and safety. The most comprehensive study to date was a retrospective study by Ma et al. (2023). This study recruited 35 patients with foreign body-related duodenal perforation and concluded that nearly 75% of the patients can be successfully treated by endoscopy alone, with a shorter hospital stay and low procedure-related complication rate [9]. In our case, the patient had successful foreign body removal via endoscopic approach with a post-procedure hospital stay of 13 days. The long length of stay can be attributed to the need for a full course of antibiotics for local peritonitis and the waiting time for a follow-up CT scan.

While there is no consensus regarding the selection criteria for endoscopic treatment for duodenal perforation specifically caused by ingested foreign body, Ansari et al. (2019) proposed that for duodenal perforation of all causes in general, endoscopic closure is more preferable in a clinically stable patient when the onset time is early (<24 hours) and the defect size is small (<3cm) [10]. In this case, our patient presented soon after symptoms onset with a stable haemodynamic condition. The perforation was sub-centimetre in size. These favourable conditions rendered endoscopic repair a reasonable option.

The authors also believe a retained intraluminal foreign body shown on preoperative imaging is another important selecting factor for endoscopic therapy as this can spare an extension of perforation from surgical intervention which may further compromise the healing of the contaminated tissue.

For post-procedure monitoring, the American Gastroenterological Association advocates a water-soluble contrast series 2-4 days after the procedure to confirm the absence of leakage before resumption of enteral feeding [11]. The usual practice in our centre is to arrange a follow-up CT scan with oral and intravenous contrast around one week after endoscopic closure to assess the perforation site and look for any nearby intra-abdominal collection.

## Conclusions

This case illustrates that endoscopic treatment of foreign body-related duodenal perforation is feasible. Careful imaging assessment and patient selection dictate the success of management. For stable patient with concealed perforations and retained intraluminal foreign body, timely endoscopic intervention spares them from major laparotomies which are inevitably associated with morbidity, reoperations and compromised quality of life.
